# Message from the new Co-Editor-in-Chief

**DOI:** 10.1038/s41514-017-0003-1

**Published:** 2017-02-15

**Authors:** Shin-ichiro Imai

**Affiliations:** Washington University School of Medicine, St Louis, MO USA

Dear our Readers,

On November 1, 2016, I became Co-Editor-in-Chief of *npj Aging and Mechanisms of Disease (npjAMD)*, with Dr. Kazuo Tsubota, who has been leading this new international journal in the field of aging research since its inauguration. I am excited to work with Dr. Tsubota, all Associate Editors and Editorial Board members, and staff members of *npjAMD* to bring this excellent journal to the next level. Additionally, on behalf of all editorial team members, I would like to welcome a new Associate Editor and two new Editorial Board members. Dr. Vera Gorbunova at University of Rochester became a new Associate Editor, and Drs. William Mair at Harvard TH Chan School of Public Health and Meng Wang at Baylor College of Medicine joined our Editorial Board. Dr. Gorbunova has been a leading figure for comparative biology of aging, particularly focusing on naked mole rat, and also publishing impressive studies on SIRT6, a mammalian sirtuin family member, and DNA damage response. Dr. Gorbunova’s expertise will surely help enrich the coverage and the quality of our Journal. Both Drs. Mair and Wang are rising stars in the field of aging research, using *C. elegans* as their favorite model organism. *npjAMD* puts an emphasis on understanding the basic mechanisms of aging, and *C. elegans* has been providing a powerful genetic tool to discover important signaling pathways and regulatory molecules for the control of aging and longevity. Drs. Mair and Wang will critically enhance the strength of our journal to cover the cutting-edge fronts of model organism-driven basic aging research.

Population aging is a serious, global challenge to everybody in the world. To achieve “productive aging”, we need to understand how and why we age at molecular, cellular, and physiological levels and find hardcore evidence-based effective solutions. With these new editorial members including myself, *npjAMD* will take significant efforts to promote aging and longevity research worldwide through nurturing close scientific communications in our growing aging research community.

Taking this opportunity, I would like to set out a couple of audacious goals for *npjAMD*: (1) To provide an active, international discussion forum to the community of aging and longevity research through our Journal, and (2) To significantly increase the number of high-quality papers published in *npjAMD*. For the former, I would like to propose to create a new section named “Discussion Forum” covering hot front lines and controversies in the field of aging and longevity research. I hope this new section will provide inspiration and insight to many researchers in the field worldwide. For the latter, I will expand my own networking efforts to tap as many people as possible through e-mails, phone calls, and meetings and conferences, and try to find out new, exciting studies in the field. Of course, I would also like to hear from our readers about their new research and findings. When you find me at any meeting, symposium, or conference, please feel free to talk to me, or email us at the editorial office. I will be happy to learn and discuss about your new findings and provide some advices in case you would be interested in submitting your manuscript to our Journal. Your ideas on how the journal should be developed will also be highly welcomed.

